# Hybrid constitutive law with machine learning for sintering of advanced ceramics

**DOI:** 10.1038/s41598-026-58322-3

**Published:** 2026-06-24

**Authors:** Baber Saleem, Peter Polak, Ran HE, Savvaki Savva, Jonathan Phillips, Jingzhe Pan

**Affiliations:** 1 School of Engineering, University of Leicester, Leicester, LE1 7RH UK; 2 Morgan Advanced Materials, Morgan Dr, Stourport-On-Severn, DY13 8DW UK

**Keywords:** Sintering deformation, Constrained sintering, Hybrid constitutive modelling, Artificial neural networks, Finite element analysis, Engineering, Materials science, Mathematics and computing

## Abstract

Predictive simulation of sintering-induced distortion remains challenging for ceramic components subjected to gravity and mechanical constraint. Classical constitutive sintering laws reproduce free densification reliably but lack the flexibility required to accurately capture stress-driven deformation within finite-element (FE) frameworks when calibrated solely from dilatometer data. This study presents a hybrid machine-learning-assisted constitutive framework for modelling constrained sintering of an industrial ceramic material. Dilatometer densification data and a gravity-loaded beam-bending experiments were obtained for the same material system, enabling simultaneous evaluation of volumetric sintering kinetics and part-level deformation. Two independently calibrated parameter sets of an Olevsky-type constitutive law reproduce densification behaviour but underpredict gravity-driven curvature (A) when applied within FE simulations, highlighting an inherent trade-off between densification fitting and deformation prediction. To overcome this limitation, the analytical volumetric strain-rate term is replaced by an artificial neural network trained directly on experimental densification data, while analytical formulations for mean and deviatoric stress response are retained. This hybrid framework decouples densification kinetics from shear-dominated deformation, enabling modulation of the effective viscous stiffness governing beam bending without compromising physical interpretability or numerical robustness. The results establish a simple, computationally efficient, and physically interpretable pathway toward predictive modelling of constrained sintering, providing a scalable foundation for industrial process optimisation and future digital-twin development.

## Introduction

Sintering is a critical densification step in advanced ceramic manufacturing, transforming porous green compacts into dense functional components through diffusion-driven mass transport. Accurate prediction of sintering-induced deformation is essential for ensuring dimensional stability, improving yield, and enabling process optimisation in high-value industrial applications. Despite extensive modelling efforts, reliable prediction of sintering distortion remains challenging because key constitutive parameters—such as viscosity, sintering stress, and microstructure evolution—are difficult to determine experimentally and often exhibit strong coupling and non-uniqueness^1–4^.

The densification-based finite element method (DFEM) was developed to address these challenges by substituting experimentally measured densification rates for explicit rheological descriptions^5–7^. DFEM has demonstrated success in predicting free and constrained sintering deformation without extensive parameter calibration. However, because it relies directly on densification data, it does not explicitly account for stress-dependent mean and deviatoric contributions that govern deformation in constrained geometries such as beams, plates, or layered assemblies^8,9^. In parallel, the master sintering curve (MSC) framework has provided an efficient route for extracting apparent activation energies and unifying densification behaviour across multiple thermal histories^10–13^. While both approaches have significantly advanced sintering simulation, they remain limited by their empirical nature and lack flexibility when applied to evolving industrial materials and complex stress states.

More recently, machine learning (ML), particularly artificial neural networks (ANNs), has emerged as a powerful tool for modelling nonlinear relationships in materials processing^14–18^. In ceramic systems, ML has been applied to predict microstructure evolution^19^, densification kinetics^20^, final density^21–23^, and shrinkage behaviour^24^. However, the integration of ML within physics-based sintering frameworks remains limited. Physics-informed and physics-based neural networks offer a promising pathway for combining experimental data with mechanistic constraints^25,26^. In the context of sintering, He et al.^27^ demonstrated a neural-network-based constitutive replacement capable of reproducing deformation in fine-grained alumina, while Ghantasala and Singh^28^ combined ML with finite-element modelling to predict deformation during pressureless sintering of additively manufactured metallic components. Although these studies highlight the potential of hybrid learning–simulation strategies, they primarily target laboratory-scale or metallic systems and do not explicitly integrate data-driven densification kinetics within a stress-decomposed constitutive framework suitable for industrial ceramics.

In our previous work^29^, we demonstrated that machine learning can be used to replace the volumetric component of a classical sintering constitutive law, where an ANN was trained to predict densification rate as a function of temperature, integral temperature, and relative density, and embedded into ABAQUS via a user subroutine. Separately, in^30^, a DFEM-based digital twin approach was developed to predict sintering distortion of an industrial Ox–Ox CMC component using densification data, without employing machine learning. While both approaches showed strong capability in predicting free-sintering behaviour, neither addressed stress-coupled deformation under constrained conditions.

The present study extends this concept to constrained sintering by introducing a hybrid ANN–constitutive framework. In this approach, the ANN is used exclusively to predict the volumetric free-sintering strain rate from experimental dilatometer data, while the analytical mean and deviatoric stress components—parameterised through viscosity constants—are explicitly retained within an Olevsky-type constitutive formulation. This partitioned strategy enables data-driven densification kinetics to be combined with a physics-based stress response within a single finite-element implementation. Unlike fully data-driven multiphysics frameworks that replace the entire constitutive description^31^, the present methodology deliberately restricts machine learning to the volumetric response, thereby preserving physical transparency of stress contributions and ensuring numerical robustness within standard finite-element solvers.

Recent studies have addressed constrained sintering using both fully coupled constitutive models and hybrid data-driven approaches. For example, Cabo Rios et al.^32^ employed a phenomenological viscoplastic framework to capture densification and gravity-induced distortion through calibrated material parameters. More recently, Manière and Hammoud^33^ proposed a hybrid FEM–deep learning framework in which elastic simulations are used as descriptors to predict distortion risk.

However, these approaches either rely on explicit constitutive parameter calibration or operate at the structural level as distortion predictors, rather than being directly integrated within a constitutive finite-element formulation. The present work addresses this gap by embedding a machine-learned constitutive response directly within the FE framework, enabling simultaneous prediction of densification and stress-driven deformation under general loading conditions.

The framework is evaluated using a gravity-loaded beam-bending experiment performed on an industrial ceramic material, for which both dilatometer densification data and part-level deformation measurements are available from the same material system. Validation focuses on the experimentally measurable curvature parameter (*A*), providing a stringent benchmark for assessing stress-driven sintering deformation under gravity. Through this approach, the study demonstrates that targeted integration of machine learning at the constitutive level can substantially improve predictive capability for constrained geometries while remaining compatible with industrial finite-element workflows.

## Methodology

### Theoretical background and the hybrid constitutive framework

Sintering of ceramics involves coupled densification and viscous flow driven by temperature-dependent diffusion mechanisms. The constitutive framework proposed by Olevsky^2^ provides a continuum description of this behaviour by expressing the total inelastic strain-rate tensor ${\dot{\varepsilon }}ij$ as the sum of deviatoric, mean, and volumetric contributions:

1 $${\dot{\varepsilon }}ij = \frac{1}{2G}S_{ij} + \frac{1}{3K}\left( {p - \sigma_{s} } \right)\delta {\mathrm{i}}j$$

where ${S}_{ij}$ is the deviatoric stress tensor, p is the mean stress, ${\upsigma}_{\mathrm{s}}$ is the sintering stress and $\updelta \mathrm{i}\mathrm{j}$ is the Kronecker delta function. The shear viscosity G and bulk viscosity K are defined as**:**

2 $${\mathrm{G}}\, = \,\eta_{s} .\rho^{{2n_{s} - 1}} ,$$

3 $${\mathrm{K}}\, = \,3. f_{s}^{2} .\eta_{b} { }.\rho^{{2n_{b} - 1}} ,$$

where ${\eta}_{s}$ and ${\eta}_{b}$ represent the material resistance to shape (shear) and volume (bulk) deformation, respectively. Both viscosities follow Arrhenius-type temperature dependencies:

4 $$\eta_{s} = C_{s1 \left( s \right)} T\exp \left( \frac{Q}{RT} \right)2d^{m} ,$$

5 $$\eta_{b} = C_{s1 \left( b \right)} T\exp \left( \frac{Q}{RT} \right)2d^{m} ,$$

where ${C}_{s1 (s)}$ and ${C}_{s1 (b)}$ are viscosity constant, Q is activation energy for the material determined using the MSC approach from ^11^, d is the grain radius, R is the universal gas constant, T is the absolute temperature (K), ${n}_{s}$ and ${n}_{b}$ are the exponential constant for shear and bulk viscosities respectively, and represents the grain evolution term with exponent m = 3.

Under free-sintering conditions, where no external stresses are applied, both the deviatoric stress ${S}_{ij}$ and the mean stress ${\upsigma}_{\mathrm{m}}$ vanish. Equation 1 therefore reduces to a purely volumetric strain-rate formulation,

6 $${\dot{\varepsilon }}_{v} = \frac{{ - \sigma_{s} }}{K},$$

where the sintering stress ${\upsigma}_{{\mathrm{s}}}$ is given by

7 $$\sigma_{s} = \frac{2\gamma }{{\zeta d}}\rho^{{N_{s} }} \left( {\frac{{\rho \left( {1 - \rho_{0} } \right)}}{{\rho_{0} \left( {1 - \rho } \right)}}} \right)^{{{\raise0.7ex\hbox{$1$} \!\mathord{\left/ {\vphantom {1 3}}\right.\kern-0pt} \!\lower0.7ex\hbox{$3$}}}} ,$$

with γ denoting the specific surface energy, ζ the sintering stress correction factor, Ns the sintering stress exponent, and ρ_0_ the initial relative density. The sintering function fs, which accounts for the porosity dependence of densification, is defined as

8 $$f_{s} = \frac{1}{{{\raise0.7ex\hbox{$5$} \!\mathord{\left/ {\vphantom {5 2}}\right.\kern-0pt} \!\lower0.7ex\hbox{$2$}}\sqrt {1 - \rho } }}.$$

Equations 1–7 form the basis of the numerical implementation used for constitutive parameter fitting and FE simulations. These relations were employed within a MATLAB-based framework to calibrate material parameters against free-sintering densification data.

The DFEM approach^5,6^ simplifies the implementation of Olevsky’s theory by replacing the analytical volumetric strain-rate term (Eq. 6) with experimentally measured densification rates obtained from dilatometry. While DFEM accurately reproduces free-sintering behaviour, it does not explicitly represent the mean and deviatoric stress contributions (Eqs. 2, 3), which become dominant under constrained sintering conditions such as beam bending.

To address this limitation, a hybrid ANN–constitutive framework was developed. In this approach, the volumetric strain rate ${\dot{\varepsilon }}_{v}$ is predicted directly by an artificial neural network trained on experimental densification data, while the mean and deviatoric stress contributions remain governed analytically by the constitutive relations. The resulting hybrid formulation is expressed as

9 $${\dot{\varepsilon }}ij = \frac{1}{2G}S_{ij} + \frac{1}{3K}p \delta {\mathrm{i}}j - \dot{\varepsilon}_{v}^{ANN} \delta {\mathrm{i}}j$$

where ${C}_{s1 (s)}$ and ${C}_{s1 (b)}$ and ${n}_{s}/{n}_{b}$ control the temperature- and density-dependent shear and bulk responses through Eqs. 2–5, thereby linking the analytical and data-driven components of the model. As a consistency check, each fitted parameter set was evaluated by computing the corresponding Poisson ratio,

10 $$\nu = \frac{{3{\text{K }} - { }2{\mathrm{G}}}}{{6{\mathrm{K}} + { }2{\mathrm{G}}}},$$

to ensure physically admissible behaviour (0 < ν < 0.5) throughout the sintering cycle.

### Experimental data

#### Temperature profiles and dilatometer densification data

The ceramic powder investigated in this study is a production-grade industrial material supplied by Morgan Advanced Materials (UK) and can be broadly classified as a 96% alumina ceramic. The powder was blended with binders, spray-dried, and uniaxially pressed into green compacts at 260 MPa using a laboratory hand press. The resulting cylindrical specimens had a length of 11–12 mm and a radius of approximately 6 mm. All dilatometer experiments were conducted at Morgan Advanced Materials.

The initial grain size of the material is approximately ~ 2 μm. Grain size measurements indicate that, during ramp-up heating under the conditions explored in the dilatometry experiments, the grain diameter remains approximately constant at ~ 2 μm. The final grain size after full sintering is approximately ~ 4 μm. Significant grain growth is therefore not observed during the ramp-heating stage considered in this study, but may occur under extended dwell conditions.

**Fig. 1:**
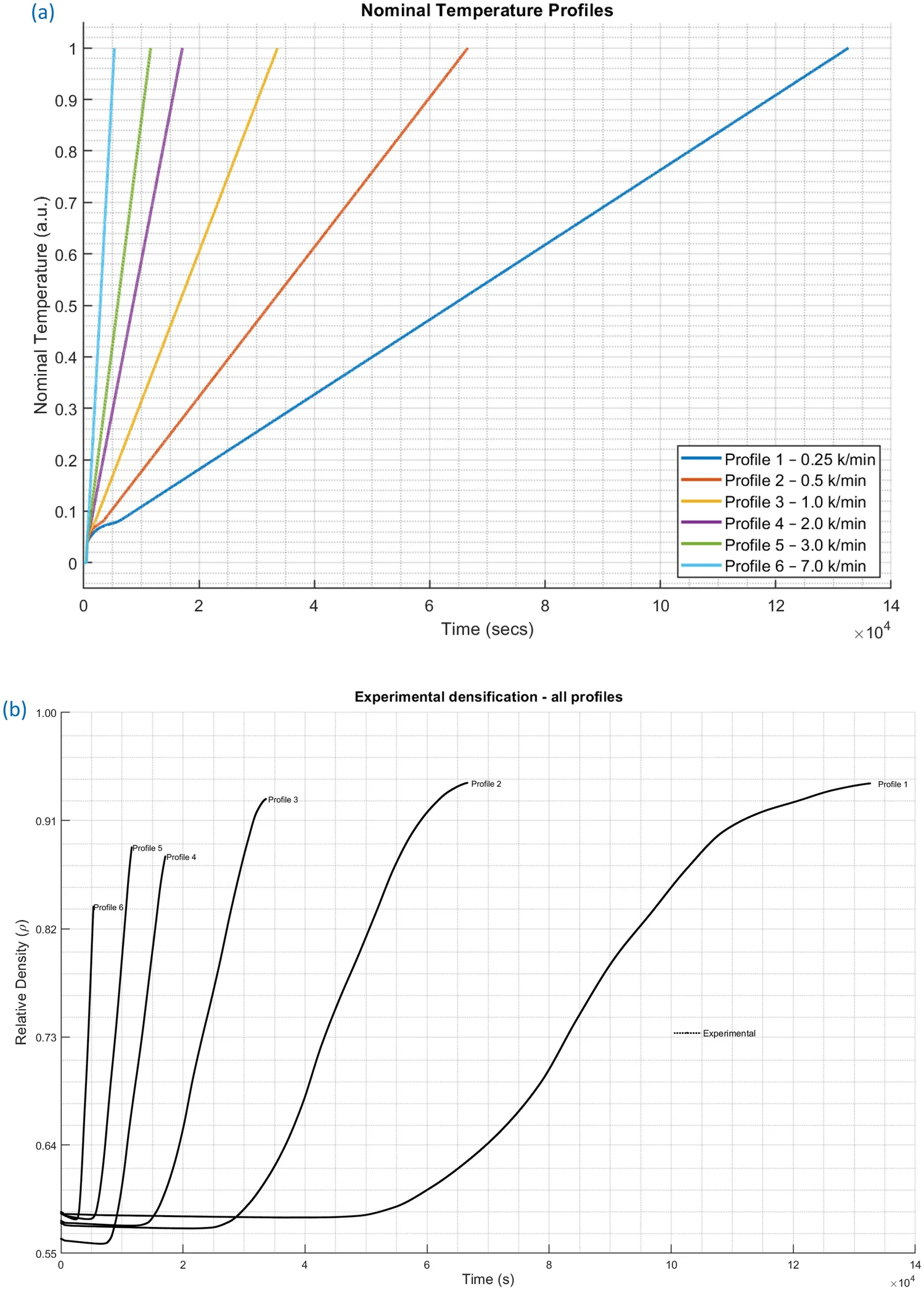
(**a**) Normalised temperature profiles for the six sintering conditions. (**b**) Experimentally measured densification curves (Profiles 1–6) showing relative density evolution with time.

Six constant heating-rate sintering profiles without dwell were employed: 0.25, 0.5, 1.0, 2.0, 3.0, and 7.0 K min^−1^. In all cases, the temperature was initially ramped rapidly to approximately 1270 K, after which the prescribed heating rate was applied until the final temperature was reached. The corresponding thermal histories are shown in Fig. 1a. Owing to confidentiality constraints associated with proprietary industrial data, temperature values are presented in normalised (nominal) form, scaled between the onset and maximum temperatures for each profile. The onset of sintering densification corresponds to a nominal temperature of approximately 0.42, while the peak temperature is defined as a nominal value of 1.0.

The initial ρ_0_ exhibited minor variation among samples, which is typical for laboratory-pressed compacts. The measured ρ_0_ values for the six heating profiles were 0.5991, 0.5899, 0.5919, 0.5753, 0.5976, and 0.5973. Relative density ρ was calculated with respect to the material’s theoretical maximum density of 3.8 g cm^−3^. The experimental volumetric strain rate, (ε̇ᵥ) was computed as $\dot{\uprho }/\uprho$.

These experimental datasets formed the basis for both constitutive-law fitting and ANN training.

#### Beam-bending experiment and FE ABAQUS setup

**Fig. 2:**
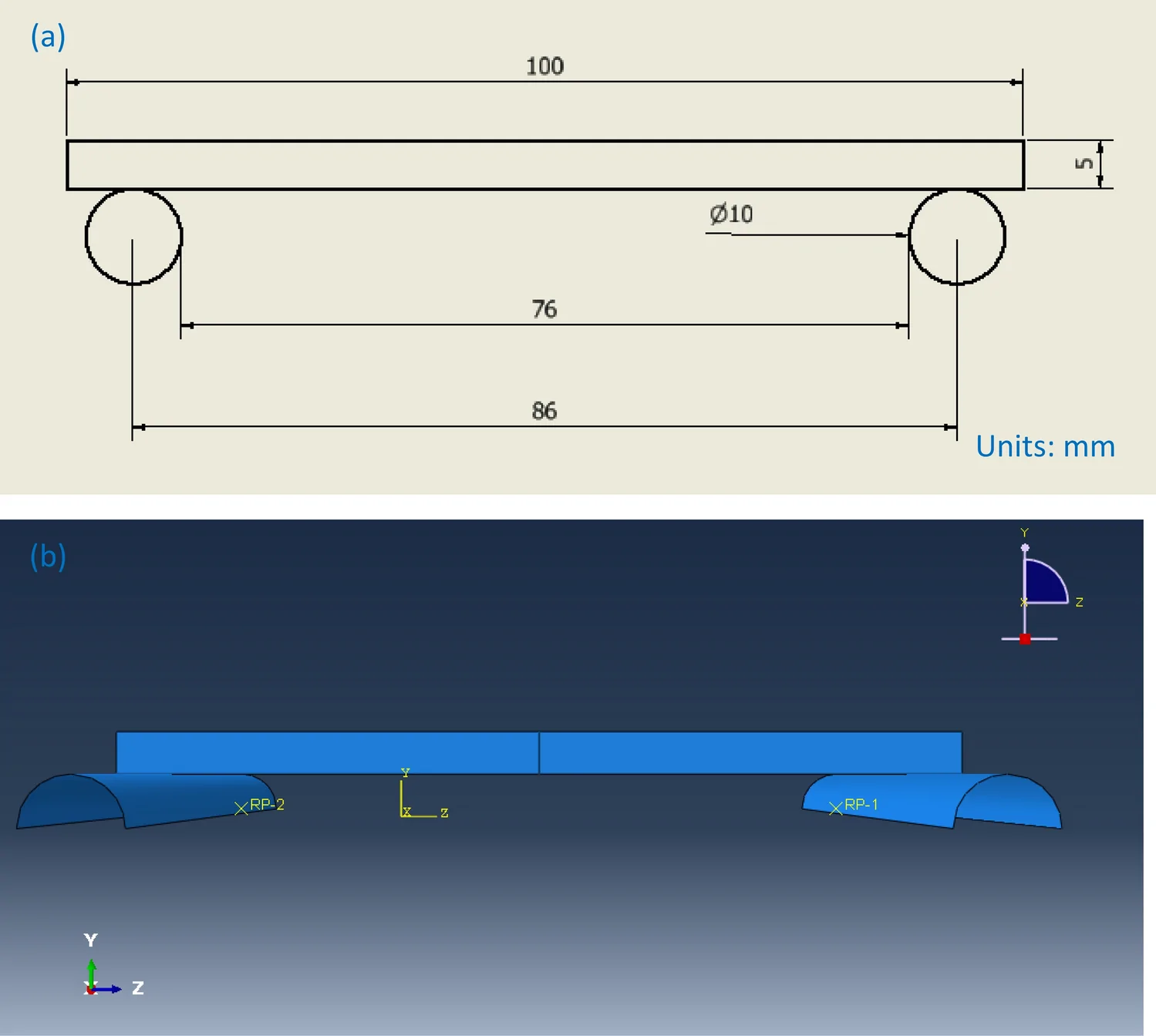
(**a**) Experimental setup for constrained beam bending during sintering. (**b**) ABAQUS model reproducing the experimental geometry, support span, and thermal cycle (1.0 K min^−1^).

To characterise constrained sintering behaviour, a beam-bending experiment was performed at a heating rate of 1.0 K min^−1^ (Profile 3—Fig. 1a). The green beam measured 100 mm in length, 5 mm in height, and 10 mm in depth, and was supported on two cylindrical rollers of 10 mm diameter, spaced 76 mm apart, giving an effective support span of 86 mm (± 43 mm from the beam mid-length), as shown in Fig. 2a. The same geometry and support configuration were reproduced in the ABAQUS/CAE 2023 FE model (Fig. 2b).

During sintering, side-view images of the beam were recorded at regular temperature intervals by Morgan Advanced Materials. For each image, the visible upper and lower beam edges were extracted and independently fitted to a quadratic function,

11 $${\mathrm{y}}_{{{\mathrm{img}}}} \, = \,{\mathrm{A}}\xi^{{2}} \, + \,{\mathrm{B}}\xi \, + \,{\mathrm{C}},$$

yielding six coefficients (Upper_A, Upper_B, Upper_C and Lower_A, Lower_B, Lower_C) together with the corresponding temperature. The supplied coefficients were filtered to remove image-acquisition noise. Based on industrial guidance, only curvature data up to approximately 1800 K were considered reliable and were used for quantitative comparison. In this representation, A describes beam curvature, B accounts for rigid-body tilt, and C represents the vertical offset in the camera coordinate system.

**Fig. 3:**
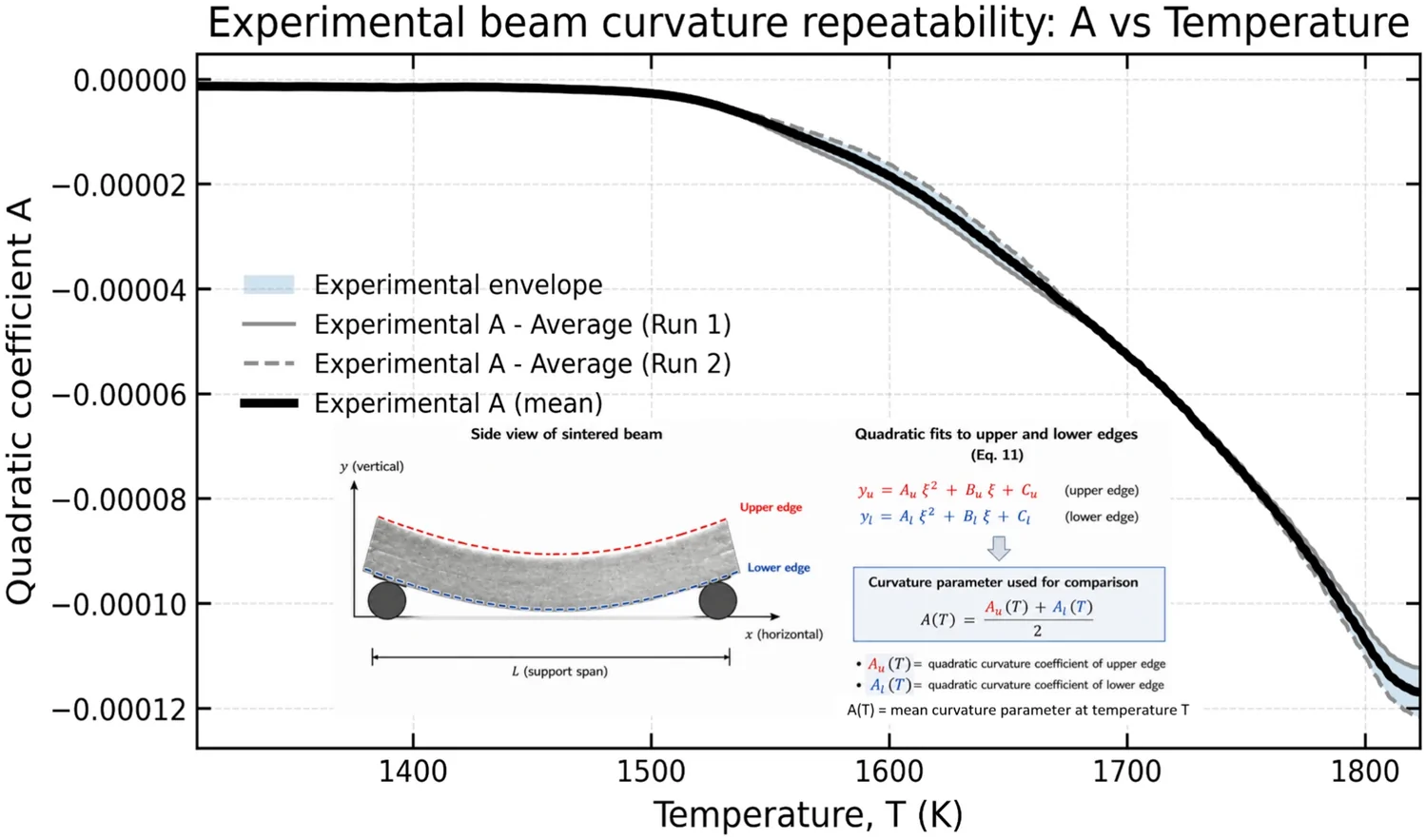
Experimental beam curvature repeatability during sintering (Profile 3, 1.0 K min^−1^). Curvature parameter A(T) is defined as the mean of the upper and lower quadratic coefficients (Eq. 11). Two independent experimental runs are shown together with their mean curvature and envelope.

Two independent beam-bending experiments (Run 1 and Run 2) were performed under identical thermal conditions to assess experimental repeatability. For each run, a curvature parameter A(T) was obtained by taking the mean of the upper and lower quadratic curvature coefficients. The mean curvature of the two runs is used for comparison with FE predictions, while Run 1, Run 2, and their envelope are shown in Fig. 3.

To enable comparison with FE results, the quadratic fits were converted from pixel units to physical coordinates. The horizontal image coordinate ξ ∈ [0, 2000] px was mapped linearly to x ∈ [− 50, + 50] mm, where [0, 2000] px corresponds to the usable beam span consistently captured within the camera field-of-view across all frames. As the image analysis provides curvature from side-view outlines only, axial shrinkage along the beam length was not quantified and is therefore excluded from the validation. The vertical scale was determined from the cold-frame image by evaluating the fitted upper and lower polynomials on a common ξ-grid and matching the average pixel separation to the known beam thickness of 5 mm. A single vertical scale factor was applied uniformly to all frames. Vertical shrinkage inferred from the fitted C coefficients was not used for validation because the absolute vertical offset is sensitive to imaging/edge-detection drift at high temperature.

All image-derived profiles were referenced to the cold-frame configuration by assigning the upper surface at mid-span (x = 0) a vertical displacement of zero. Rigid-body rotation was removed by re-referencing the profiles to the beam supports (x =  ± 43 mm), yielding a support-referenced deflection field that isolates bending deformation.

The FE model of the beam-bending experiment was constructed in ABAQUS/CAE 2023 using first the original constitutive law and subsequently the hybrid ANN–constitutive framework (Eq. 9), implemented via a user-defined CREEP subroutine. The beam geometry and support span matched the experimental configuration and were discretised using quadratic 20-noded brick elements (C3D20R), with five elements through the beam thickness and depth. The supports were modelled as analytical rigid cylinders, each assigned a reference point with fully fixed (encastre) boundary conditions. Surface-to-surface contact was defined between the beam underside (slave) and the rigid supports (master), assuming frictionless interaction.

A uniform gravitational acceleration of 9.81 m s^−2^ was applied to account for self-weight, and the experimental temperature–time profile corresponding to 1.0 K min^−1^ was imposed as a spatially uniform thermal load. For comparison with experimental data, nodal vertical displacements along the top surface of the FE beam were extracted at each output temperature, mapped onto the same axial coordinate system, and fitted using the same quadratic form (Eq. 11) as applied to the experimental images. The same support-referencing procedure was used to remove rigid-body rotation, and only the resulting curvature parameter A was retained for comparison.

#### Activation-energy determination (master sintering curve)

The apparent activation energy (Q) of the material was determined using the MSC method with the shape-function approach^11^, following the same procedure adopted in our previous study^29^. The MSC variable Θ was computed numerically as

12 $$\Theta = \mathop \int \limits_{0}^{t} \frac{1}{T}\exp \left( { - \frac{Q}{KT}} \right)\; {\mathrm{dt}}$$

where T is the absolute temperature, and t is the sintering time. Each experimental heating profile was processed iteratively by varying Q, and the corresponding densification curves were mapped onto a common master curve of relative density ρ versus ln(Θ).

**Fig. 4:**
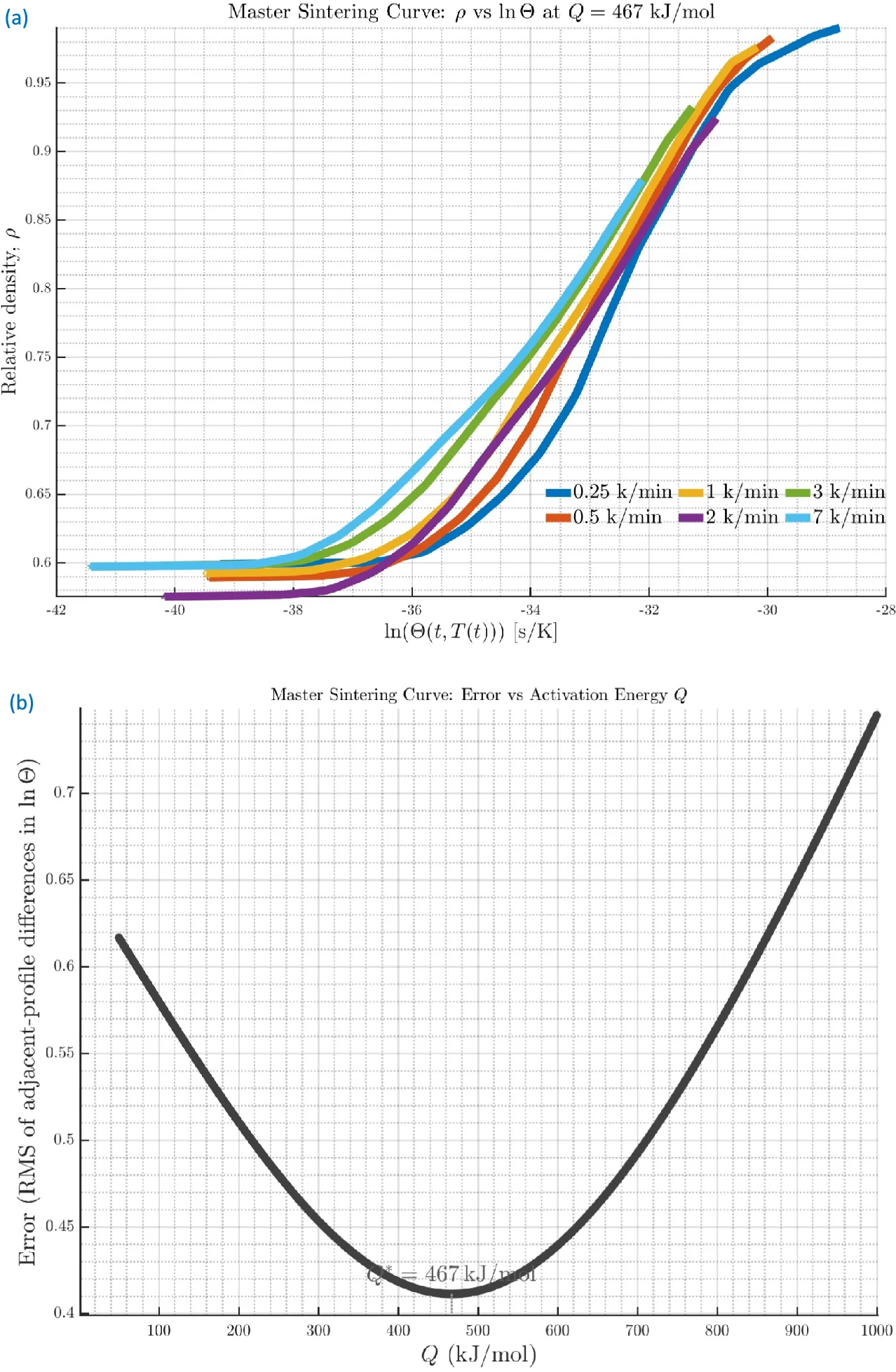
(**a**) Master sintering curve constructed using the MSC method. (**b**) Variation of root-mean-square error (RMSE) with activation energy Q, used to identify the optimal value.

The quality of curve collapse was quantified using a root-mean-square error (RMSE) criterion, calculated from the differences in ln(Θ) between adjacent heating profiles evaluated on a common ρ-grid. The value of Q corresponding to the minimum RMSE was selected as the apparent Q governing densification for the material system. The MSC construction and RMSE-based selection procedure are illustrated in Fig. 4a, b, respectively.

The Q obtained from the MSC analysis was not used directly as an input to the ANN. Instead, it was employed to generate the synthetic dataset used during the initial (pre-training) phase of ANN development, ensuring that the theoretical training data remained physically consistent with the experimentally derived densification kinetics and the calibrated constitutive parameter sets.

### Constitutive-law fitting

The sintering response of the beam material was calibrated using the original constitutive law^37^ (Eq. 1) and its hybrid extension. In the first step, the original constitutive law was fitted directly to experimental densification data obtained from dilatometer measurements (Fig. 1b). The viscosity constant Cs1 and the stress exponent n were treated as fitting parameters and explored over broad ranges (Cs1 = 10^2^–10^18^, n = 1–20) to capture physically admissible sintering behaviour.

The parameter exploration was restricted to Cs1 and n, as these directly control the viscous response through the bulk and shear viscosity terms and therefore dominate both densification kinetics and deformation behaviour. Other parameters (Table 1) were held constant to reduce the dimensionality of the fitting problem and to maintain physical consistency. In particular, the Q was fixed based on the master sintering curve (Fig. 4), while the gas constant is a known physical constant. The sintering stress parameter ζ was fixed at 1.1951, corresponding to a physically reasonable sintering stress of approximately 1 MPa under the conditions considered. Remaining parameters (e.g., Ns, γ) were retained as standard values consistent with oxide ceramic systems.

**Table 1:** Material parameters for the two best-performing parameter sets (PS1a and PS1b) identified from fitting the original constitutive law to experimental densification data. All remaining parameters were held constant, and a constant grain size (≈ 2 µm) measured during ramp-up heating was used in all simulations.

Parameter set (PS)	${C}_{s1 (s)}$ Pa s	${C}_{s1 \left(b\right)}$ Pa s	Q J mol^−1^	${n}_{b}$	${n}_{s}$	ξ	Γ	m	dm	Ns
1a	7.79 × 10^1^⁰	7.79 × 10^1^⁰	467,000	5.75	5.75	1.1951	1	3	2 × 10^−^⁶	1
1b	8.47 × 10^10^	8.47 × 10^10^	467,000	5.67	5.67	1.1951	1	3	2 × 10^−^⁶	1

As highlighted in our previous study^29^, this fitting problem is non-unique, with several hundred parameter combinations providing comparably good agreement with the densification profiles. From this admissible set, two independently calibrated parameter combinations were selected based on their ability to simultaneously reproduce both the dilatometer densification behaviour and the beam-bending response. These selected parameter sets define a feasible constitutive parameter space for modelling constrained sintering under gravity and are summarised in Table 1.

Grain size remains approximately constant (~ 2 μm) during ramp-up heating under the conditions considered, as described in "Temperature profiles and dilatometer densification data" section. Grain-growth effects are therefore neglected during constitutive parameter fitting.

While the original constitutive law provided a reasonable description of densification, its volumetric strain-rate response is analytically prescribed and remains fixed once calibrated. This limits its transferability when applied to constrained sintering conditions. To address this limitation, a hybrid constitutive framework was introduced in which the analytical volumetric strain-rate term was replaced by an ANN-based representation (Eq. 9), trained using experimental densification data. The analytical stress-dependent formulations were retained, allowing the hybrid model to improve volumetric response prediction while preserving the established deviatoric behaviour.

### Artificial neural network model

#### Architecture and training

**Fig. 5:**
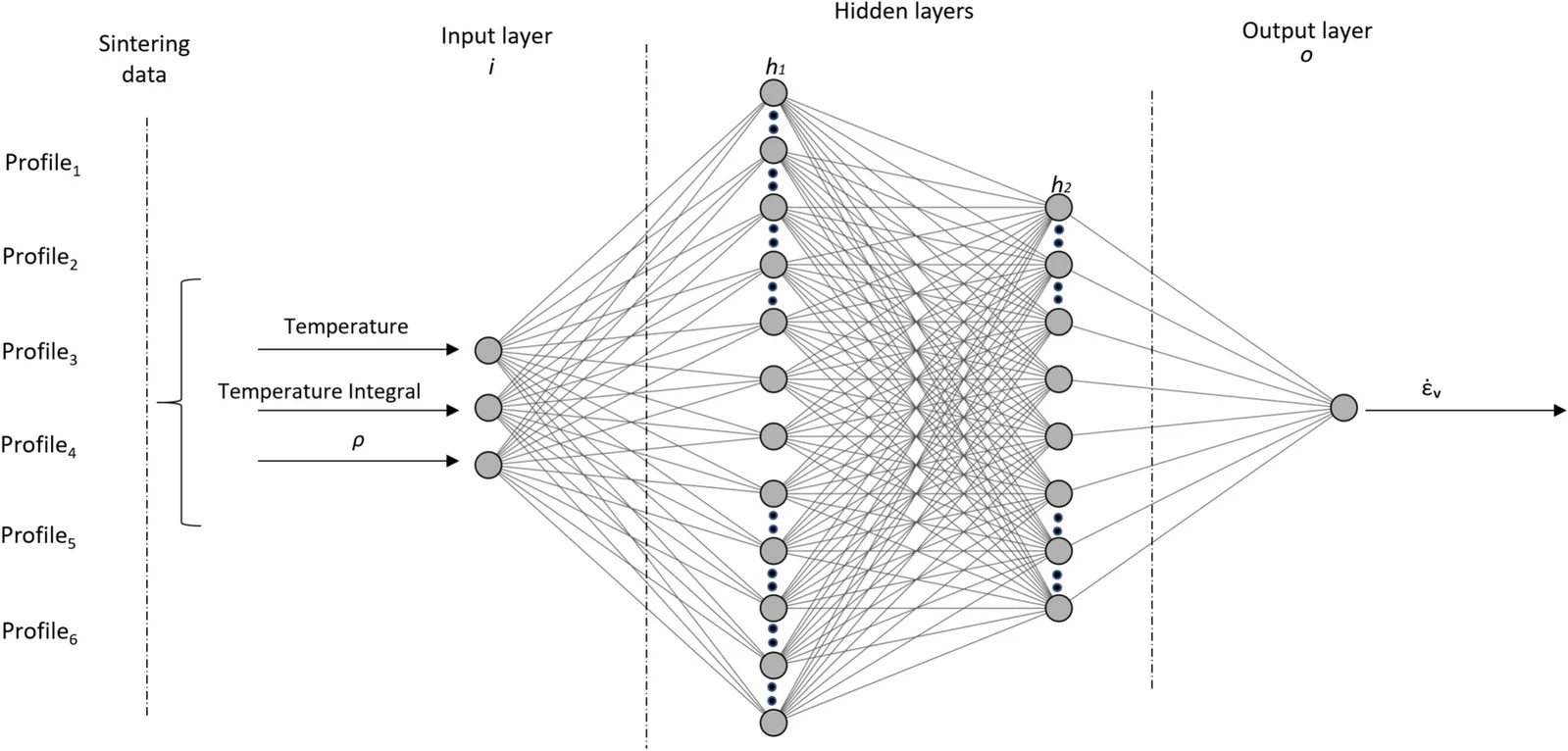
Architectural representation of the artificial neural network used in this study.

An ANN was developed to predict the volumetric strain rate (${{\dot{\upvarepsilon }}_{\mathrm{v}}}^{\mathrm{A}\mathrm{N}\mathrm{N}}$) used in the hybrid constitutive formulation (Eq. 9). The ANN architecture (Fig. 5) comprised three input neurons representing temperature *T*, integral temperature ∫T dt, and relative density *ρ*, derived from six experimental heating profiles (Fig. 1a).

The inclusion of the ∫T dt term is intended to capture the influence of thermal history on densification behaviour. While classical constitutive formulations typically express densification rate as a function of temperature and density, previous work^31^ demonstrated that deformation behaviour cannot be fully described without incorporating thermal history effects. In that study, the temperature rate (Ṫ) was introduced through a temperature-rate-dependent viscosity term to improve predictive capability.

Following the same physical reasoning, the present study employs the ∫T dt as a cumulative measure of thermal exposure. Unlike instantaneous temperature rate, this formulation captures the full heating trajectory, which is more appropriate for conventional sintering conditions. This allows the ANN-based model to account for history-dependent densification behaviour in a physically consistent manner.

The network included two hidden layers with 64 and 32 neurons, employing LeakyReLU and ReLU activation functions, respectively, followed by a single linear output neuron corresponding to ${{\dot{\upvarepsilon }}_{\mathrm{v}}}^{\mathrm{A}\mathrm{N}\mathrm{N}}.$

Training followed a physics-informed two-step procedure, consistent with our previous work^27,29^. This strategy is designed to ensure that the ANN remains physically consistent while effectively utilising limited experimental data.

In the first stage, the ANN was pre-trained using a large synthetic dataset (322,000 data points) generated from the Olevsky constitutive law (Eq. 1), using an activation energy *Q* = 467 kJ mol^−1^ and the fitted parameter set (Table 1). The dataset was evaluated over a wide range of temperature, relative density, and stress conditions, including gravity-induced loading. This stage enables the network to learn the fundamental relationships governing densification behaviour, thereby embedding the underlying physics of the constitutive model within the ANN.

In the second stage, the pre-trained ANN was fine-tuned using the six-profile experimental dilatometer dataset (approximately 15,000 data points per profile). This step allows the model to correct discrepancies in the analytical formulation and accurately capture the actual material response.

This combined approach ensures that the ANN is not purely data-driven, but instead represents a physics-informed model, improving robustness, convergence, and generalisation. All inputs and outputs were standardised using their respective mean (μ) and standard deviation (σ). Training was performed using the Adam optimiser with a learning rate of 1 × 10^−3^ and a batch size of 5, with early stopping based on validation MAE. The final network weights, biases, and scaling parameters were exported as plain-text files for direct use within ABAQUS via a FORTRAN-based CREEP subroutine.

#### ANN-based volumetric strain-rate formulation

The trained ANN was used to provide predictions of the volumetric strain rate ${{\dot{\varepsilon }}_{v}}^{ANN}$ as a function of *T*, ∫T dt, and *ρ*. In the hybrid constitutive framework, this ANN-predicted volumetric strain rate replaces the analytical volumetric term in the constitutive law (Eq. 9), enabling data-driven representation of densification behaviour during sintering. The implementation of ${{\dot{\varepsilon }}_{v}}^{ANN}$ within the FE framework is described next.

### Implementation of hybrid model

The hybrid constitutive model defined by Eq. 9 was implemented in an ABAQUS FORTRAN CREEP subroutine, building on the framework introduced in^29^. The implementation embeds a complete ANN forward pass within the subroutine while retaining analytical formulations for the mean and deviatoric stress components of the modified Olevsky model. All constitutive parameters are applied uniformly over the full temperature range, with no temperature-dependent switching of material functions.

At the start of the analysis, an initialisation routine (INIT_ANN_DATA) reads the trained ANN weights, biases, scaling parameters, and the pre-computed temperature integral (∫T dt) corresponding to the imposed thermal cycle from external text files and stores them in memory for use throughout the simulation. At each increment, the CREEP subroutine retrieves the T and time increment Δt from *ABAQUS*, computes the current ρ from the accumulated volumetric strain (ESW), and accesses the corresponding ∫T dt value indexed by the increment number (KINC). These three quantities—[T, ∫T dt, ρ]—form the ANN input vector (Fig. 5).

All ANN inputs are standardised using the same scaling parameters employed during training. The scaled inputs are propagated through the trained network using LeakyReLU and ReLU activation functions in the first and second hidden layers, respectively, followed by a linear output neuron to obtain ${{\dot{\varepsilon }}_{v}}^{ANN}$. After inverse scaling, the predicted volumetric strain rate is converted into an incremental volumetric strain contribution and assigned to DESWA(1). In parallel, the deviatoric and mean strain-rate contributions are evaluated analytically and using Eq. 2–5, based on the shear and bulk viscosity parameters (${C}_{s1 (s)}, {C}_{s1 \left(b\right)},$ ${n}_{s}$, ${n}_{b}$) and the current state variables (STATEV). Temperature and density-dependent quantities, including the σ_s_, and fs, are updated at every increment.

Selected STATEVs, including ρ, σ_s_ (Eq. 7), effective viscosities (Eqs. 2–3), and the ANN-predicted volumetric strain-rate increment (DESWA(1)), were stored during the simulation to facilitate monitoring of internal variables and numerical consistency. Further details on the implementation of DECRA and DESWA parameters in user-defined creep subroutines are provided in the ABAQUS user subroutines reference manual^38^.

All FE simulations were performed using quadratic brick elements with five elements through the beam thickness and depth. For this discretisation, a complete beam sintering simulation required approximately 65–70 min of wall-clock time when executed on a single CPU core. The additional computational cost associated with the ANN forward pass within the CREEP subroutine was negligible compared to the overall FE solution time.

## Results

### Densification fitting using original constitutive law

**Fig. 6:**
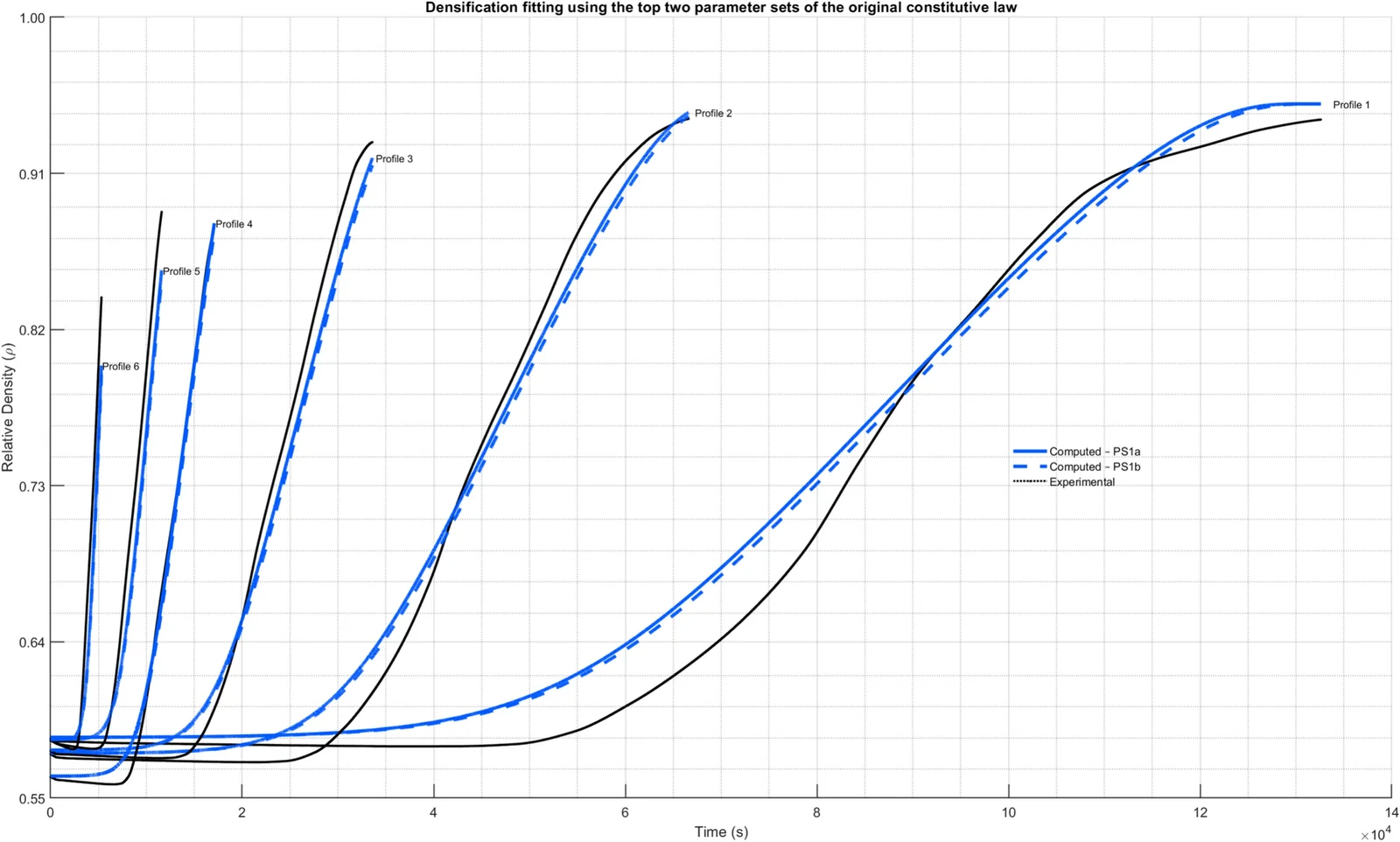
Comparison of experimental and computed densification curves for all six heating profiles using the original constitutive law with the two best-performing parameter sets.

The densification behaviour predicted using the original constitutive law was compared with experimental dilatometer data for all six heating profiles (Fig. 6). A wide constitutive parameter space was explored, yielding multiple admissible solutions, reflecting the non-unique nature of constitutive parameter fitting when based solely on densification data (Fig. 1b). Model performance was evaluated quantitatively using the MAE in ρ.

From this parameter space, the two best-performing parameter combinations, denoted PS1a and PS1b, were identified based on their agreement with the experimental densification curves (Table 1). PS1a achieved the lowest overall MAE of 0.01388, with per-profile MAEs of 0.01640, 0.01254, 0.01285, 0.00592, 0.01750, and 0.01806 for Profiles 1–6, respectively. PS1b yielded a slightly higher overall MAE of 0.01506, with corresponding per-profile MAEs of 0.01566, 0.01350, 0.01462, 0.00758, 0.01941, and 0.01956. For Profile 3, which corresponds to the beam-bending experiment, both parameter sets show comparable accuracy, with MAEs of 0.01285 for PS1a and 0.01462 for PS1b, supporting their subsequent use in constrained sintering simulations. The corresponding densification curves are shown in Fig. 6.

### Finite-element prediction of beam curvature evolution

**Fig. 7:**
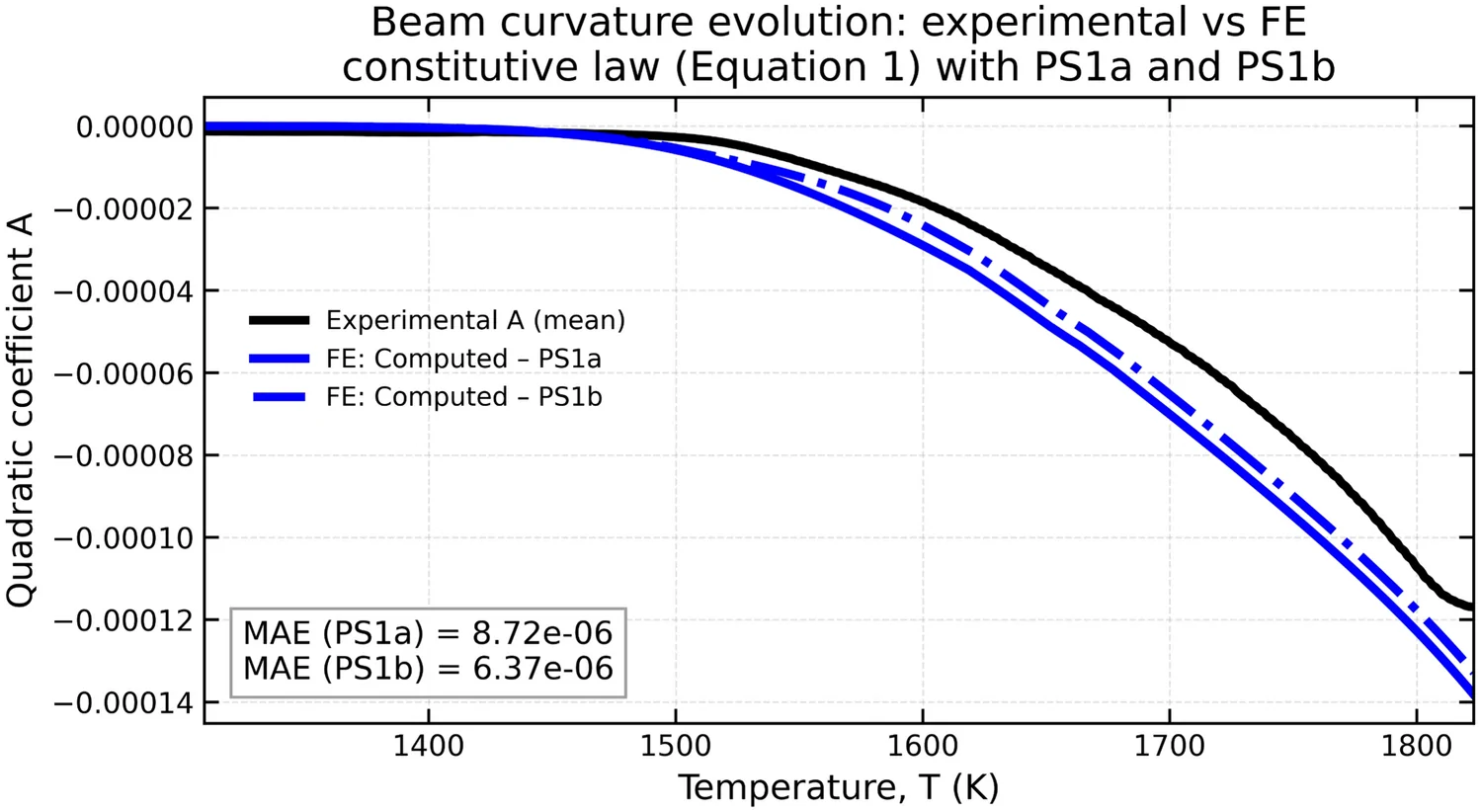
Experimental and FE-predicted evolution of the beam curvature parameter A with temperature using the original constitutive law (Eq. 1) with parameter sets PS1a and PS1b.

Figure 7 compares the experimentally measured evolution of the beam curvature parameter A with FE predictions obtained using the original constitutive law (Eq. 1) with the two best-performing parameter sets, PS1a and PS1b, for the 1.0 K min^−1^ heating profile.

Both FE simulations capture the onset of curvature at low temperatures and reproduce the overall trend of increasing bending with temperature. As temperature increases, the FE predictions slightly underpredict the magnitude of curvature relative to the experimental response; however, the deviation remains moderate over the full temperature range considered.

Quantitatively, PS1a yields a curvature MAE of 8.72 × 10⁻⁶, while PS1b provides a marginally lower MAE of 6.37 × 10⁻⁶. These values are computed relative to the mean experimental curvature shown in Fig. 3. These results demonstrate that multiple admissible parameter sets derived from densification fitting can produce similar curvature predictions, highlighting the non-unique mapping between densification-based constitutive parameters and macroscopic distortion behaviour.

### Independent verification of ANN training (outside ABAQUS)

Although the FE predictions obtained using PS1a and PS1b reproduced the overall experimental beam-curvature trend, quantitative agreement with the measured curvature evolution remained limited. To isolate model-form accuracy from FE discretisation and boundary-condition effects, the ANN was therefore independently verified outside the FE environment prior to its integration into ABAQUS. This verification was performed using a standalone Python implementation, in which the trained ANN weights and biases were used in a forward-pass evaluation to generate volumetric strain-rate predictions directly from the experimental temperature–density histories (Fig. 1a, b).

**Fig. 8:**
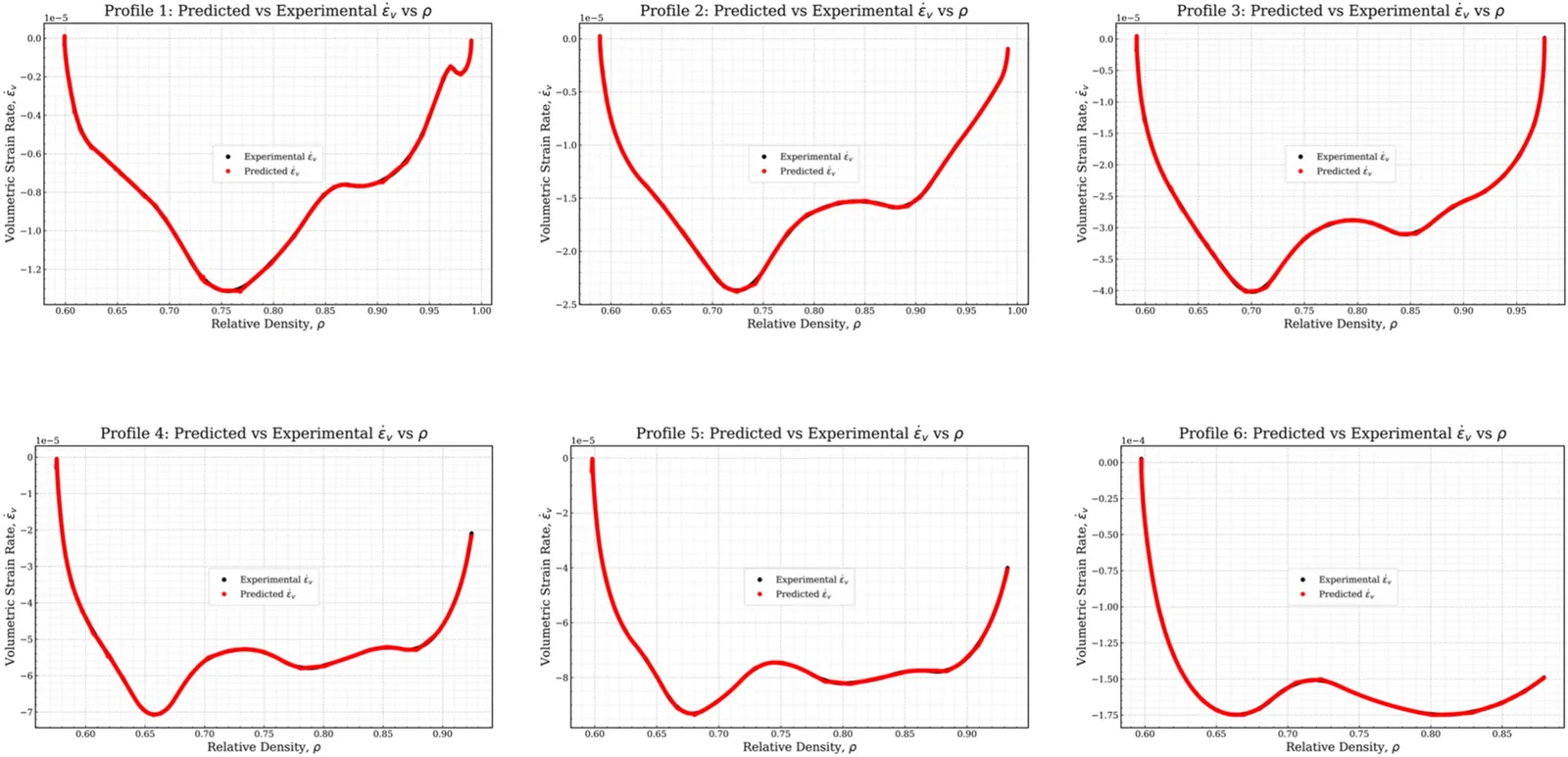
Independent ANN verification: predicted and experimental volumetric strain rate ${\dot{\varepsilon }}_{v}$ as a function of relative density for six heating profiles.

Figure 8 compares the ANN-predicted volumetric strain rate, ${{\dot{\varepsilon }}_{v}}^{ANN}$, with experimentally derived values as a function of relative density ρ for all six heating profiles. Across the full density range, the ANN accurately reproduces the characteristic density-dependent evolution of ${\dot{\varepsilon }}_{v}$, including acceleration at intermediate densities and saturation near full density.

The MAE values for the six heating profiles were 1.74 × 10^−8^(0.25 K min^−1^), 2.58 × 10^−8^ (0.5 K min^−1^), 4.21 × 10^−8^ (1.0 K min^−1^, beam profile) 5.98 × 10^−8^ (2.0 K min^−1^), 1.15 × 10^−7^ (3.0 K min^−1^), and 1.62 × 10^−7^ (7.0 K min^−1^). While the error increases with heating rate, reflecting stronger nonlinear densification behaviour, it remains consistently low across all profiles.

These results confirm that the ANN provides a high-fidelity representation of volumetric densification behaviour independent of finite-element implementation, establishing a robust foundation for its subsequent coupling within the FE framework.

### Integration of ANN-predicted densification kinetics into FE framework

Following independent verification, the trained ANN was integrated into the FE framework via the ABAQUS *CREEP* subroutine (Eq. 9). Initial verification was performed using a simple cube geometry discretised with a single quadratic 20-noded brick element (C3D20R), ensuring numerical stability and correct coupling of the ANN-predicted volumetric strain rate under thermal loading.

**Fig. 9:**
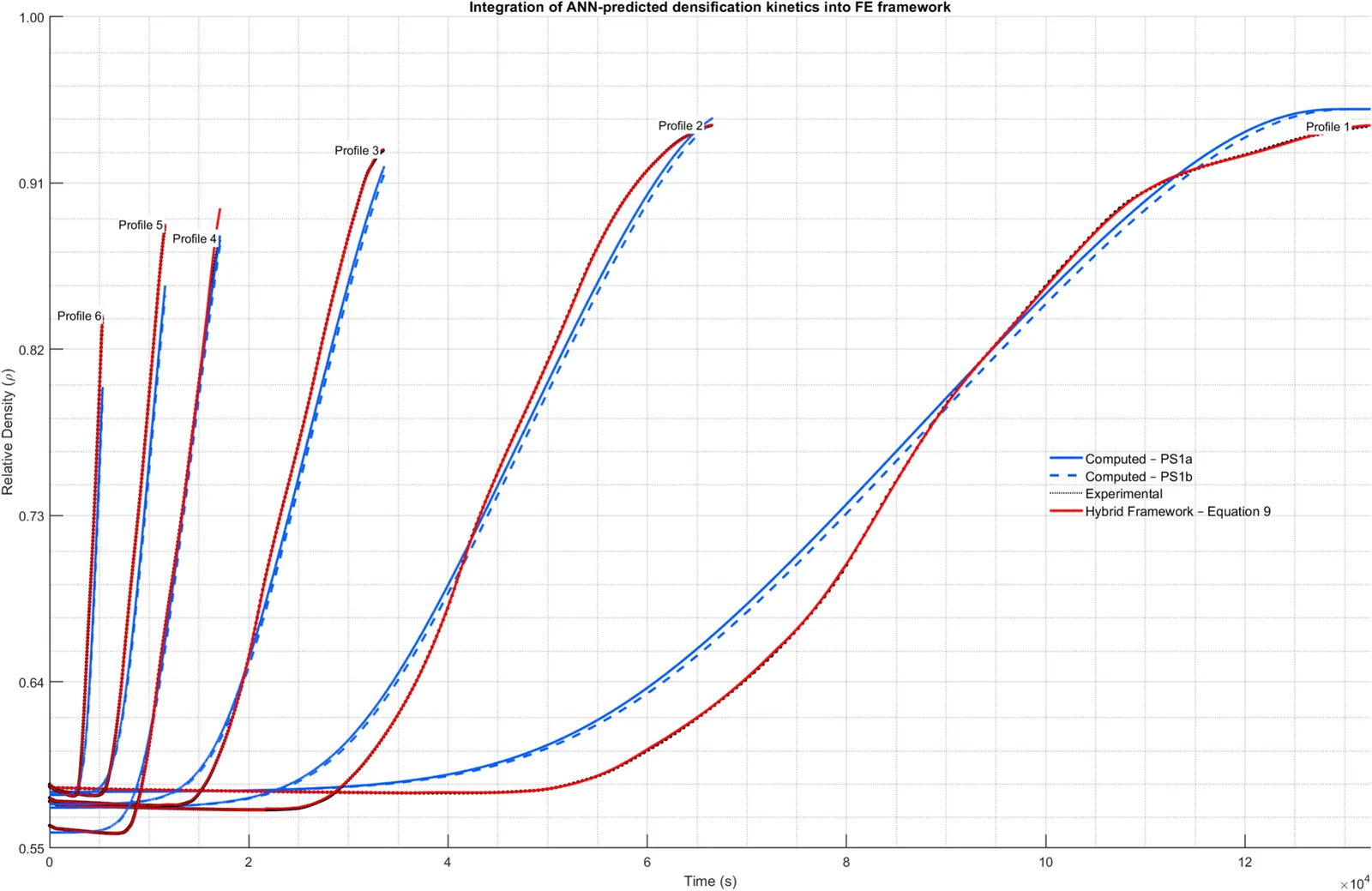
Comparison of experimental densification curves with finite-element predictions obtained using the analytical constitutive law and the ANN-driven hybrid framework for all six heating profiles.

The ANN-driven FE simulations accurately reproduced the experimental densification histories for all six heating profiles. Figure 9 compares the experimentally measured ρ(t) evolution with FE predictions obtained using the analytical constitutive formulation based on parameter sets PS1a and PS1b, and the ANN-based hybrid framework using Eq. 9.

Across all profiles, the hybrid framework provides consistently improved agreement with the experimental ρ(t) evolution compared with the analytical constitutive law, particularly at intermediate and high densities. This demonstrates robust numerical integration of the ANN-predicted ${{\dot{\varepsilon }}_{v}}^{ANN}$ within the FE environment and confirms the capability of the hybrid approach to capture densification kinetics under coupled thermal–mechanical conditions.

### Beam-bending validation of the hybrid framework

**Fig. 10:**
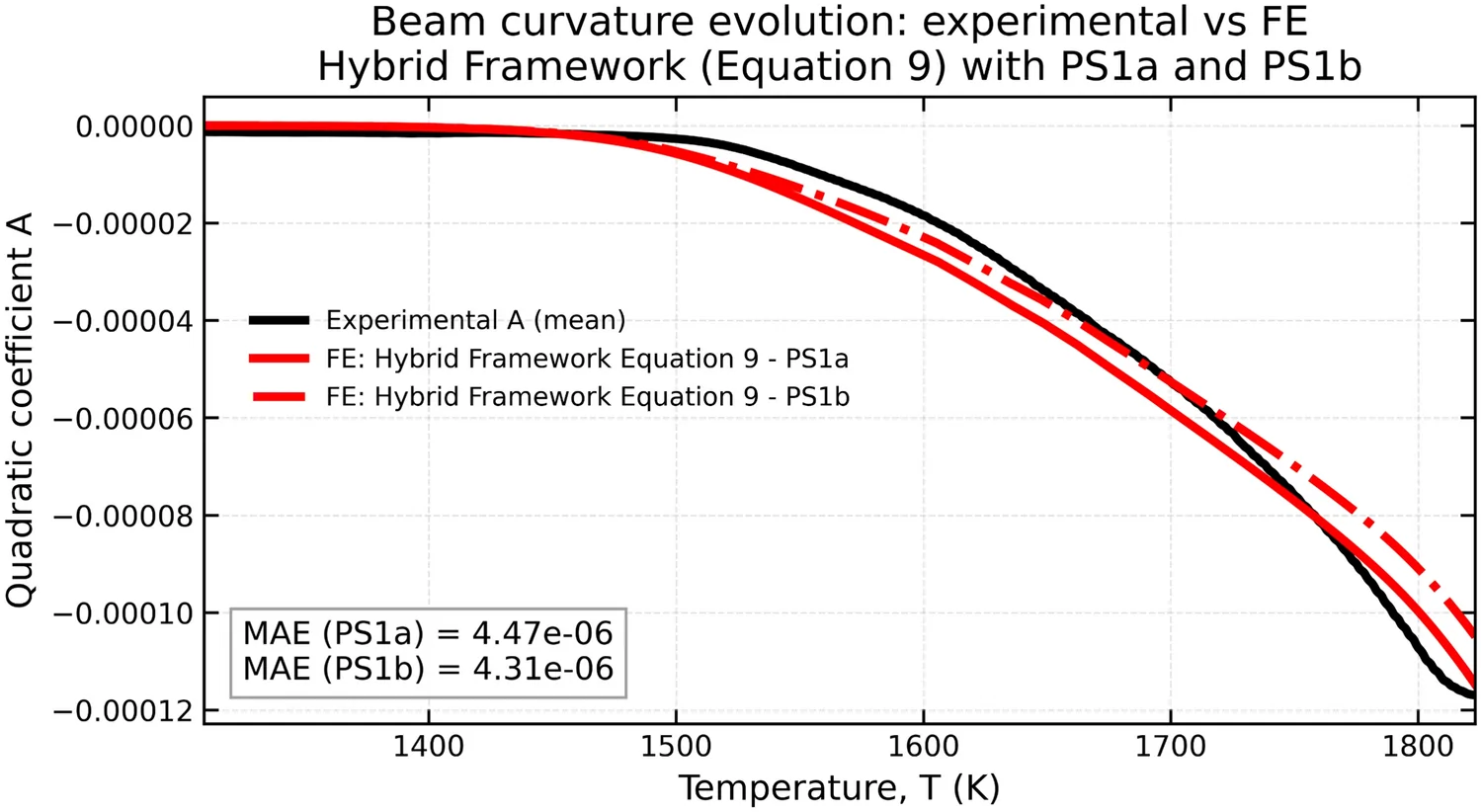
Comparison of experimentally measured beam curvature evolution with FE predictions obtained using the hybrid framework (Eq. 9) for parameter sets PS1a and PS1b.

The hybrid framework (Eq. 9) was validated by comparing FE predictions of the beam curvature parameter A(T) with experimentally measured curvature evolution obtained from beam-bending experiments (Fig. 10). In both the experimental post-processing and FE analysis, curvature was defined consistently as the average of the upper and lower A quadratic coefficients.

As described in the Methodology, axial shrinkage is not represented in the experimental outline measurements and is therefore excluded from the validation. In addition, through-thickness shrinkage inferred from the intercept term of the quadratic fits is not considered due to its limited experimental reliability. Consequently, validation is performed exclusively using curvature-based metrics.

Figure 10 compares the experimentally measured curvature evolution with FE predictions obtained using the ANN-assisted hybrid framework (Eq. 9) for two independently calibrated parameter sets, PS1a and PS1b. Both parameter sets reproduce the onset and progressive development of beam curvature with good agreement over the reliable experimental temperature range of approximately 1300–1823.15 K. The predicted curvature magnitude and trend closely follow the experimental response, particularly during the high-temperature regime where curvature increases rapidly.

Quantitatively, the curvature MAE is reduced from 8.72 × 10⁻⁶ and 6.37 × 10⁻⁶ for PS1a and PS1b, respectively, using the analytical formulation, to 4.47 × 10⁻⁶ and 4.31 × 10⁻⁶ with the ANN-assisted hybrid framework. This corresponds to an error reduction of approximately 49% for PS1a and 32% for PS1b, demonstrating a substantial improvement in predictive accuracy.

## Discussion

This study extends our earlier investigations on sintering deformation by critically examining the limitations of classical constitutive descriptions when applied to gravity-driven, constrained sintering. While previous DFEM-based studies^5,6,30^ and our earlier ML-assisted framework^29^ focused primarily on free sintering behaviour, the present work directly targets distortion arising from self-weight and mechanical constraint, which remains a critical challenge in industrial ceramic manufacturing. Even small inaccuracies in densification kinetics or stress evolution can lead to unacceptable dimensional deviations and component rejection. FE analysis provides a natural framework for linking densification, gravity, and evolving material properties; however, its predictive capability is fundamentally limited by the underlying constitutive formulation (Eq. 1).

It is important to clarify that the influence of gravity as an external load, and the associated limitations of classical constitutive formulations in predicting constrained sintering deformation, are well established in the literature^1–4,8,9^. The present work does not aim to identify this limitation as a new finding. Rather, the gravity-loaded beam configuration is introduced as a stringent and physically meaningful benchmark, in which known deficiencies in the constitutive formulation are amplified into measurable curvature A (Fig. 3). This enables a direct and quantitative assessment of model fidelity and provides a robust basis for evaluating the effectiveness of the proposed hybrid framework.

Calibration of the analytical constitutive law (Eq. 1) using dilatometer densification data yields approximate agreement with experimental relative-density evolution across all heating profiles (Fig. 7). Nevertheless, as highlighted in our previous work^29^, this calibration is inherently non-unique: multiple combinations of viscosity constants (Cs1) and stress exponents (n) reproduce near-identical densification curves while producing markedly different deformation responses under constraint. Similar observations have been reported in recent ML-assisted densification studies^22,23^, where accurate reproduction of densification kinetics alone does not guarantee correct prediction of macroscopic distortion. The present study mitigates this limitation by combining densification data with part-level deformation data (Fig. 3) obtained from the same industrial material system. While non-uniqueness persists if densification is considered in isolation, the inclusion of repeatable beam curvature evolution (Fig. 3) introduces an independent mechanical constraint that substantially reduces the admissible parameter space and exposes deficiencies in purely densification-based calibration.

Application of the analytical constitutive law (Eq. 1), using the independently calibrated parameter sets PS1a and PS1b (Table 1), reproduces densification kinetics with reasonable accuracy but fails to accurately predict gravity-driven beam curvature (Fig. 8). In both cases, the analytical model underpredicts the magnitude of curvature relative to experiment. Mechanistically, this reflects a limitation of the analytical formulation in representing the coupled interaction between volumetric densification and shear-dominated deformation under self-weight, once the constitutive parameters are fixed by free-sintering densification data. Further fine-tuning of constitutive parameters can reduce the curvature error; however, such adjustments lead to deterioration in the quality of the densification fits shown in Fig. 7. This highlights an inherent trade-off in constitutive calibration, whereby no single analytical parameter set can simultaneously reproduce both experimental densification behaviour and macroscopic curvature evolution with high fidelity.

**Fig. 11:**
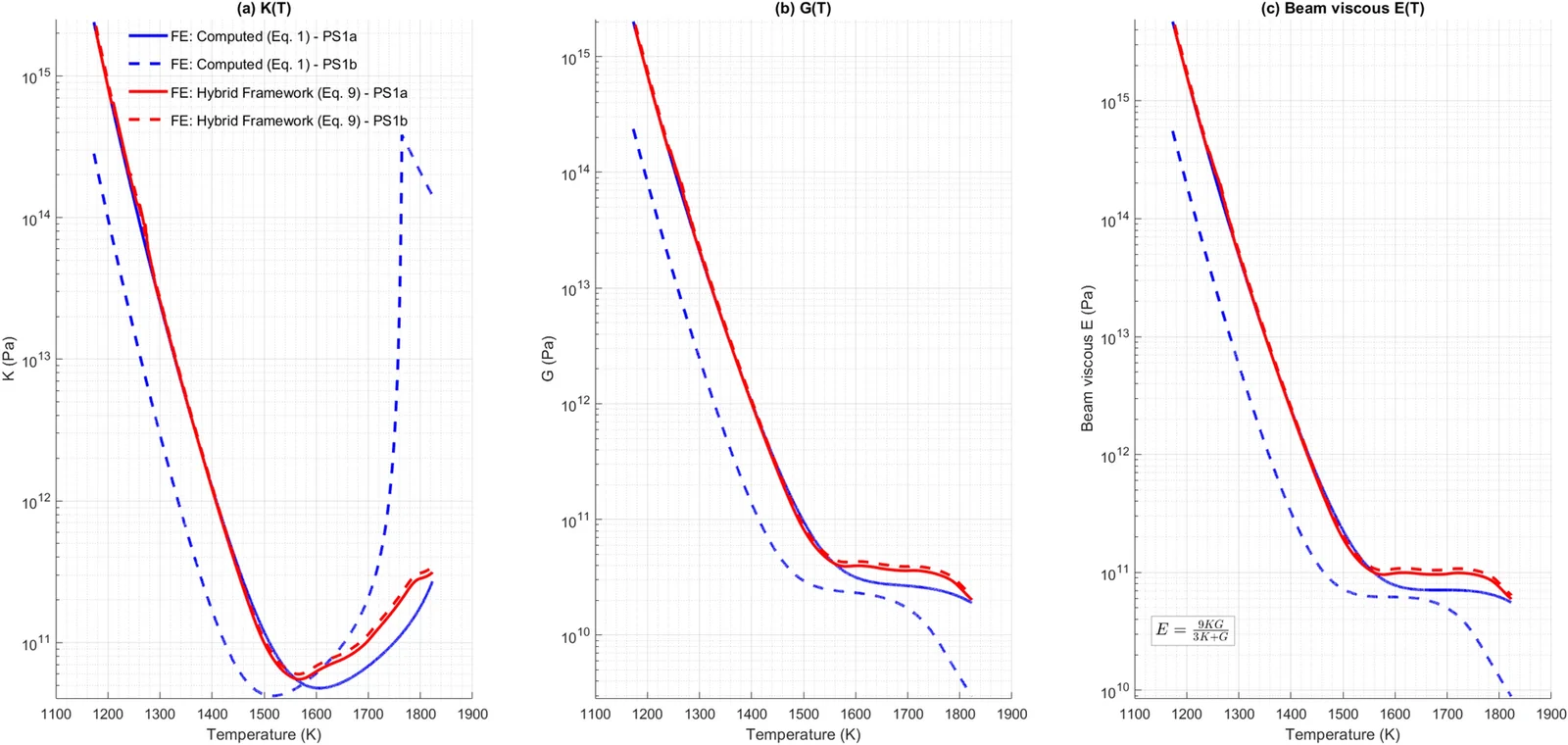
Temperature-dependent viscous moduli extracted from FE simulations for the experimental beam (Profile P3, 1 K min^−1^): (**a**) bulk viscosity modulus K(T)—Eq. 3, (**b**) shear viscosity modulus G(T)—Eq. 2, and (**c**) effective beam viscous modulus E(T) = 9KG/(3K + G), comparing the analytical constitutive law (Eq. 1) and the hybrid framework (Eq. 9) for parameter sets PS1a and PS1b.

This limitation is further clarified by analysis of the temperature-dependent viscous moduli extracted directly from the FE simulations (Fig. 11), where the effective beam viscous modulus is computed consistently from the stress and strain-rate fields for both the analytical constitutive law (Eq. 1) and the hybrid formulation (Eq. 9). For the analytical formulation, the resulting effective beam viscous modulus E(T) = 9KG/(3K + G), evolves in a manner that does not provide sufficient flexibility to reconcile gravity-driven bending with densification behaviour once the parameters are fixed by free-sintering calibration. Consequently, the analytical model systematically underpredicts the magnitude of beam curvature (Fig. 8), despite reproducing the correct qualitative trend. This behaviour reflects the prescribed nature of the analytical volumetric strain-rate term (Eq. 6), which constrains the coupled evolution of volumetric densification and shear-dominated deformation under self-weight, thereby limiting quantitative agreement with experimental curvature evolution.

The hybrid framework (Eq. 9) overcomes this limitation by decoupling volumetric densification kinetics from the analytical stress response. By replacing the analytical volumetric strain-rate term with an ANN-predicted volumetric strain rate trained directly on experimental dilatometer data, densification behaviour is represented independently of the parameters governing bulk and shear viscosities. Independent forward-pass verification confirms that the ANN accurately reproduces experimental densification kinetics, while in-ABAQUS simulations demonstrate numerically stable and accurate densification under gravity loading (Fig. 10).

Quantitative analysis of viscous moduli provides a direct mechanistic link between the hybrid framework and the observed curvature response. For PS1a, the hybrid framework produces a modest reduction in mean effective beam viscous modulus relative to the analytical law (− 8.68% over the full temperature range and − 8.60% for T ≤ T_ref_ = 1455 K), with a small increase above T_ref_ (+ 6.07%). In contrast, for PS1b the hybrid formulation predicts a substantially higher viscous stiffness than the analytical law, with mean E(T) increasing by approximately 718% over the full temperature range and by approximately 725% for T ≤ T_ref_. These contrasting stiffness shifts demonstrate that curvature differences arise primarily from how the framework modulates the effective viscous stiffness through K(T) and G(T), rather than from geometric or loading assumptions.

It is noted that the two parameter sets PS1a and PS1b (Table 1) correspond to closely spaced values of the viscosity prefactor Cs1, differing by less than 10%, and therefore represent physically admissible variations within a single material system. The observed differences in E(T) do not indicate distinct material behaviours, but arise from the nonlinear coupling between bulk and shear viscosities and the ANN-modified volumetric response within the hybrid formulation.

Validation against beam-bending experiment (Fig. 12) confirms the effectiveness and robustness of the hybrid framework. Using the curvature parameter, A(T) as the sole quantitative validation metric—consistent with experimental reliability and geometric mapping—the hybrid model reproduces both the evolution and magnitude of beam curvature with substantially reduced error relative to the analytical constitutive law. Importantly, this improvement is observed consistently across both independently calibrated parameter sets, PS1a and PS1b. These results confirm that the hybrid framework provides a significantly more accurate representation of beam curvature evolution during sintering than the analytical constitutive law, while maintaining consistency across independently calibrated parameter sets. Although axial and through-thickness shrinkage are excluded from quantitative validation due to experimental uncertainty, the hybrid FE simulations naturally predict coupled deformation modes within a single, physically consistent framework. At the final sintering temperature, the hybrid model predicts a mid-span thickness reduction of the same order as that inferred experimentally (approximately 9–11% predicted versus approximately 8–9% inferred experimentally), corresponding to a difference of around 1–2 percentage points. While this comparison is not intended as a rigorous validation, it demonstrates that the hybrid framework captures bending-driven deformation without introducing non-physical thickness evolution, and highlights its intrinsic capability to represent coupled axial, bending, and through-thickness responses once reliable experimental measurements become available.

The robustness of the proposed framework is further supported by its consistent performance across independently calibrated parameter sets. As shown in "Constitutive-law fitting" section, multiple combinations of constitutive parameters (Cs1, n) can reproduce similar densification behaviour, reflecting the inherent non-uniqueness of classical calibration. Despite this, the hybrid ANN framework yields consistent improvement in curvature prediction for both PS1a and PS1b, demonstrating that the model response is not sensitive to a specific parameter choice. This provides practical evidence that the ANN-based formulation remains stable and does not introduce bias from the pretraining stage, but instead captures material behaviour in a manner guided by experimental data.

Recent studies on constrained sintering provide useful context for positioning the present framework. Phenomenological approaches such as Cabo Rios et al.^32^ employ fully coupled viscoplastic constitutive models to capture densification and gravity-induced deformation through calibrated material parameters. While physically grounded, these approaches rely on multi-parameter identification and can exhibit sensitivity to calibration when constrained by limited experimental data. In contrast, the present framework avoids explicit calibration of the volumetric response by learning densification behaviour directly from experimental data, while retaining the analytical stress formulation.

More recent hybrid approaches, such as Manière and Hammoud^33^, combine FEM and machine learning at the structural level, using simulation-derived descriptors to predict distortion risk. Such approaches are effective for rapid screening but do not explicitly represent constitutive behaviour and are not embedded within the finite-element formulation.

The key distinction of the present work is the integration of machine learning at the constitutive level, enabling direct prediction of volumetric strain rate within the FE solver while preserving the analytical description of stress-driven deformation. This allows consistent coupling between densification and stress evolution, providing full-field predictive capability without requiring extensive constitutive parameter fitting. In this sense, the framework advances beyond existing approaches by combining physical interpretability with data-driven accuracy within a unified FE implementation.

Compared with earlier video-imaging-based FE approaches^39^, which relied on repeated manual calibration and extensive FE iterations, the present framework offers improved robustness and industrial practicality. Unlike prior DFEM-based studies^5,6,30^, which replaced volumetric densification without explicitly addressing stress-driven deformation, the hybrid approach selectively applies machine learning only to the volumetric response while retaining analytical bulk and shear stress formulations. Fully data-driven ANN-based constitutive replacements^31^ achieve high predictive accuracy but typically require large training datasets and extended training times that can span weeks to months, and they incur significant computational overhead during FE execution. In contrast, the present partitioned hybrid strategy preserves physical transparency, ensures numerical robustness, and remains computationally efficient within standard FE solvers, making it well suited for industrial adoption in predictive modelling of constrained sintering processes.

The activation energy Q (≈ 467 kJ mol^−1^) derived from the master sintering curve (Fig. 4) constrains the constitutive parameters (Table 1) by implicitly assuming a dominant diffusion mechanism over the entire sintering cycle. For oxide ceramics, this magnitude of Q is most commonly associated with grain-boundary diffusion^34–36^, and the approximately constant grain size observed during ramp-up heating (∼2 μm) supports this interpretation for the present experiments.

However, sintering is governed by multiple diffusion mechanisms that may coexist or evolve with temperature, density, and time. Fixing a single *Q* therefore represents an inherent simplification of the analytical constitutive law (Eq. 1), limiting its ability to accommodate transitions in sintering mechanisms or microstructural evolution. This limitation becomes particularly relevant for industrial sintering cycles involving extended dwell, where significant grain growth is expected and incorporation of a grain growth model would be required for accurate prediction of sintering kinetics and deformation.

In the present study, the framework is intentionally restricted to ramp-up heating conditions without dwell, consistent with the available experimental dataset and current industrial requirements. Under these conditions, grain size remains approximately constant and its influence on viscosity evolution is limited. Accordingly, the ANN-based volumetric strain-rate model (Eq. 9) is trained exclusively on ramp-heating data and is therefore valid within this domain. Application to dwell-inclusive cycles would constitute extrapolation beyond the training space and may not be physically reliable. If extension to such conditions is required, the framework can be adapted through retraining of the ANN using dwell-inclusive experimental data, together with incorporation of a physically consistent grain growth model. This approach is supported by our previous work^27^, where grain size evolution was explicitly coupled with densification behaviour within a physics-based formulation.

A formal sensitivity analysis of constitutive parameters and the ANN training strategy is not included in the present study and is identified as an important direction for future work to further quantify parameter influence and model uncertainty.

Although gravity-driven beam bending represents an extreme case not typically encountered in routine manufacturing, it provides a stringent benchmark for evaluating sintering models under coupled densification and deformation. As shown in Fig. 11, the hybrid framework (Eq. 9) reproduces the experimental curvature evolution with low MAE, yielding values of 4.47 × 10⁻⁶ for PS1a and 4.31 × 10⁻⁶ for PS1b, and demonstrating consistent accuracy across independently calibrated parameter sets. This successful reproduction of curvature, together with accurate densification kinetics (Fig. 10), confirms that the proposed hybrid framework offers a simple, computationally efficient, and physically interpretable pathway toward predictive modelling of constrained sintering. By combining dilatometer data, part-level deformation measurements, physics-based FE modelling, and targeted machine learning, the framework establishes a scalable foundation for industrial sintering optimisation and the future development of digital twins for ceramic manufacturing.

## Conclusions

This study demonstrates that classical analytical constitutive sintering laws, when calibrated solely against dilatometer densification data, are intrinsically limited in their ability to predict gravity-driven deformation during constrained sintering. Although multiple parameter sets can reproduce experimental densification histories with comparable accuracy, no single analytical calibration simultaneously captures both densification kinetics and macroscopic curvature evolution under self-weight. This limitation originates from the analytically prescribed volumetric strain-rate formulation, which remains inherently coupled to bulk and shear responses once the constitutive parameters are fixed.

To overcome this constraint, a hybrid ANN–constitutive framework was developed in which the volumetric free-sintering strain rate is replaced by an ANN trained directly on experimental densification data, while analytical formulations governing mean and deviatoric stress response are retained. This partitioned strategy decouples densification kinetics from shear-dominated deformation, enabling the effective viscous stiffness governing beam bending to evolve independently of the parameters used to fit densification behaviour.

Implementation of the hybrid framework within ABAQUS demonstrates numerically stable and computationally efficient coupling of data-driven densification kinetics with physics-based stress response. Quantitative validation against a gravity-loaded beam-bending experiment shows a substantial reduction in curvature error relative to the analytical constitutive law, with MAE reduced to 4.47 × 10^−6^ and 4.31 × 10^−6^ for two independently calibrated parameter sets. The repeatability of the experimental beam curvature (Fig. 3) further supports the robustness of the validation dataset. The consistent improvement across parameter sets confirms that the enhanced predictive capability arises from the hybrid formulation itself rather than from parameter tuning.

By selectively applying machine learning only to the volumetric response, the proposed framework preserves physical interpretability, avoids the computational overhead and training burden associated with fully data-driven constitutive replacements^31^, and remains compatible with standard industrial FE workflows. The results establish a robust and scalable pathway toward predictive modelling of constrained sintering and provide a practical foundation for the future development of digital twins for ceramic manufacturing as richer deformation datasets become available.

## Data Availability

The experimental datasets used in this study were obtained from Morgan Advanced Materials (UK) under confidentiality agreements and therefore cannot be publicly shared. Processed data supporting the findings of this study are available from the corresponding author upon reasonable request. The computational framework, ANN parameters (weights and biases), and implementation details used for the simulations can also be provided upon request.
